# Impact of Multiple Sclerosis and Its Association with Depression: An Analytical Case-Control Investigation

**DOI:** 10.3390/healthcare10112218

**Published:** 2022-11-04

**Authors:** Francisco Javier Ruiz-Sánchez, Maria do Rosário Martins, Salete Soares, Carlos Romero-Morales, Daniel López-López, Juan Gómez-Salgado, Ana María Jiménez-Cebrián

**Affiliations:** 1 Research, Health and Podiatry Group, Department of Health Sciences, Faculty of Nursing and Podiatry, Industrial Campus of Ferrol, Universidade da Coruña, 15403 Ferrol, Spain; 2 UICISA:E, Escola Superior de Saúde, Instituto Politécnico de Viana do Castelo, Rua D. Moisés Alves Pinho 190, 4900-314 Viana do Castelo, Portugal; 3 Faculty of Sport Sciences, Universidad Europea de Madrid, Villaviciosa de Odón, 28670 Madrid, Spain; 4 Department of Sociology, Social Work and Public Health, Faculty of Labor Sciences, University of Huelva, 21007 Huelva, Spain; 5 Safety and Health Postgraduate Program, Universidad Espíritu Santo, Guayaquil 092301, Ecuador; 6 Nursing and Podiatry Department, Faculty of Health Sciences, Instituto de Investigación Biomédica de Málaga (IBIMA), University of Malaga, 29071 Malaga, Spain

**Keywords:** multiple sclerosis, depression, Beck depression inventory

## Abstract

Multiple sclerosis (MS) is a neurological, chronic, inflammatory, and progressive disease with musculoskeletal problems and neurodegenerative disorders that causes worsening of the health status of patients. The aim of this study was to determine the level of depression in MS patients compared to a population of healthy subjects. The established sample size was 116 subjects matched with the same age, sex, and body mass index. The subjects were recruited from different multiple sclerosis associations and neurology clinics in different public health areas (case group *n* = 58) and healthy subjects from the same locality (control group *n* = 58). The scores and categories of the Beck Depression Inventory (BDI) in its Spanish version were collected. There was a clear statistically significant difference (*p* < 0.05) in the BDI scores between both groups. As a result, we found that the subjects with MS presented worse results with BDI = 9.52 ± 7.70 points compared to the healthy subjects with a BDI score = 5.03 ± 5.14. Within the BDI categories, there were statistically significant differences (*p* < 0.001), which were greater for the MS group. Depression is a dangerous factor for MS patients, being a trigger for a poorer quality of life.

## 1. Introduction

Multiple sclerosis (MS) is a chronic inflammatory neurodegenerative disease of the central nervous system. MS presents sexual dimorphism showing importance in the incidence and severity of the disease. The female sex shows a higher incidence of the disease and the male sex shows faster clinical progression [1,2]. Numerous epidemiological studies in the Spanish population indicate that it is a region of medium-high prevalence of the disease throughout the whole country. The cases have been increasing progressively and have reached 80–180 cases per 100,000 inhabitants with the frequency being higher in women [3].

This pathology is characterized by the spread of spatio-temporal lesions, with frequent exacerbations and remissions [4]. In addition to psychomotor signs and symptoms, demyelination has psychological and psychiatric consequences, presenting depressive symptoms in 50% of people with MS [5,6,7].

The discovery of biomarkers in the blood is a fundamental tool for predicting, diagnosing, and monitoring the efficacy of depression treatment [8]. Biomarkers are present in depressive patients with autoimmune diseases, in whom, in turn, there is a development of the proinflammatory state of cytokines IL-1β, IL-6, and TNF-α [9]. Recent studies have identified that the inflammatory process is closely related to the neurodegenerative pathways associated with depression, with proinflammatory cytokines being important in its pathophysiology [10]. In autonomic diseases such as MS, proinflammatory cytokine production triggers episodes of depression [11]. Another way to measure depression-related biomarkers is through saliva where the anti-inflammatory cytokine IL-10 is significantly associated with depression severity in MS patients [12].

The diagnosis of depression in people with MS is complex as it can have a multifactorial etiology and can be associated with clinical manifestations including euphoria, anxiety, emotional lability, and psychosis [4]. A series of atypical symptoms also must be taken into account, such as peripheral facial paralysis, painless optic neuritis, and encephalopathy [13]. Histological inflammatory changes are also implicated in the etiology [14]. The state of depression represents one of the main determinants of quality of life in MS and can lead to suicidal intent [15,16] as it is related to the intake of interferon alpha and beta [17]. In addition, within the clinical picture, there are motor disorders (spasticity, ataxia, foot drop), sensory disorders (paresthesia, pain), cerebral disorders (dysarthria, gait ataxia, fear, lack of coordination of limbs), alterations of the cranial nerves (decreased visual acuity, facial muscle weakness), disorders of the autonomic nervous system (urinary and intestinal incontinence), and cognitive disorders (decreased attention) [18,19]. A study using magnetic resonance imaging (MRI) in 46 acute MS patients showed that there was a significant correlation between frontal periventricular or non-periventricular white matter lesions and psychopathological conditions such as depression [20].

Cases of depression in subjects with MS are higher in women, tending to decrease with increasing age, with 16.7% of women and 13.1% of men being affected [21]. Depression is significantly related to education, and 89.9% of MS subjects in a recent sample had mild to severe depressive symptoms [22]. There is a significant relationship between the type of MS and depression, where subjects with progressive relapsing MS have a higher risk (100%) of developing depression [23]. Likewise, there were higher depression scores in subjects with relapsing–remitting and secondary progressive MS [24].

However, there are few studies on the Spanish population that evaluate the significance of the resulting levels of depression in its three domains (affective, behavioral, and cognitive) and anxiety in patients with multiple sclerosis. Therefore, after reviewing the published literature, we found that levels of depression have not been described in previous studies comparing subjects with MS and healthy subjects. The objective of this study is to help to improve their well-being and quality of life.

Finally, the study tries to ascertain relative depression risk in people with multiple sclerosis and healthy subjects, thus making it a case-control study.

## 2. Materials and Methods

### 2.1. Design and Sample

To develop this study, a total sample of 116 subjects was recruited for an analytical, observational, and multicenter study of cases and controls carried out in different MS associations in the province of Malaga and Granada and in the neurology area of the “Hospital de Serrania de Ronda”. This research was performed according to the criteria of the Strengthening the Reporting of Observational Studies in Epidemiology guidelines [25].

The subjects who participated were patients with MS (case group *n* = 58) and without MS (control group *n* = 58) with similar socio-economic status. The patients were recruited using a convenience sampling method. Multiple sclerosis patients were taken from reference associations and healthy subjects from the same locality as the cases.

The subjects with MS were chosen once their association and neurologist had informed them of the study on depression and quality of life and they had decided to participate voluntarily.

The inclusion criteria were as follows: Between 18 and 88 years, of both sexes, able to walk, and who authorized their participation in the signing of the consent form.

The exclusion criteria of the subjects were another neurodegenerative disease other than MS, cognitive impairment, and severe mental disorder (measured with the Expanded Disability Status Scale (EDSS)). Cases and controls were matched for age, gender, and BMI.

### 2.2. Sample Size Calculation

To calculate the sample size, we used Epidat 4.2 software (Epidemiology Service of the General Directorate of Public Health of the Consellería de Sanidade (Xunta de Galicia) with the support of the Pan American Health Organization (PAHO-WHO) and the University CES of Colombia, thus obtaining specific levels of trust, power, and groups of equal size. A sample of 102 participants (51 per group) was obtained with a confidence level of 70%, an odds ratio of 2.0, a power of 0.80, and an exposure value of 66.67% for the MS group and 50% in the control group. Finally, for operational and safety reasons, a total of 58 patients (in each group) were used in this study.

### 2.3. Procedure

Data collection included general health questions associated with demographic variables (age, weight, height, sex, and BMI) and comorbid characteristics such as diabetes, arterial hypertension/hypotension, and ischemic heart disease. In addition, health questions related to the disease were recorded, such as the type of MS, degree of spasticity, years diagnosed, and medication administered.

Next, the participants were given the Spanish version of the Beck Depression Inventory (BDI) questionnaire [26,27,28]. This questionnaire was validated and translated into Spanish and is one of the instruments used to establish depression levels [29,30]. This version of the instrument consists of a total of 21 items with four alternatives each, which are classified from 0 to 3 points, giving a possible total of 63 points. The results obtained are measured in four ranges: The first category goes from 0 to 13 points (no signs of depression), the second from 14 to 19 points (mild depression), the third from 20 to 28 points (moderate depression), and the fourth from 29 to 63 points (severe depression). This instrument is one of the fastest and most efficient for the correct assessment of signs of depression with a Cronbach’s alpha coefficient of 0.889 [31].

### 2.4. Ethics Procedure

A favorable assessment was obtained from the Ethics Committee of the University of Malaga (CEUMA) with registration number 32–2021-H and the Ethics Committee of the Provincial Research of Malaga with code 5002V01. Likewise, the participants in this study completed and signed an informed consent form detailing the entire procedure and the protection of their data. This study respected all the ethical principles for experimentation and clinical research in humans of the Declaration of Helsinki (World Medical Association) and other organizations [32].

### 2.5. Statistics

SPSS 25.0v software (IBM Corp., Armonk, NY, USA) was used to perform the statistical analysis, reporting an alpha error of 0.05 for a confidence interval (CI) of 95%.

The normality of the quantitative data was demonstrated using the Kolmogorov–Smirnov test showing a *p*-value of less than 0.05 and was described as mean ± standard deviation (SD) and range (minimum–maximum). The contrasts were compared with Student’s *t*-test or the Mann–Whitney U test for independent samples. The differences between both groups were contrasted with the Chi square test (BDI category).

## 3. Results

The data obtained showed a normal distribution (*p* > 0.05) except in the ranges of depression. The investigation was completed with a sample of 102 subjects divided into *n* = 51 for the case group and *n* = 51 for the control group, being divided and matched according to age, sex, and BMI. As can be seen in Table 1, there were no statistically significant differences (*p* > 0.05) between both groups for the descriptive data.

All the participants (*n* = 116) presented the characteristics described in Table 1. It can be seen that the years of evolution of MS were high according to the mean (12.55 ± 8.53), and the type of MS that was most present in the study was remittent–recurring (93.1%).

Table 2 shows a statistically significant difference (*p* < 0.05) in the BDI scores between both groups. The highest scores correspond to subjects with MS (BDI = 9.52 ± 7.70 points) and the lowest scores for the control group (BDI = 5.03 ± 5.14). Statistically significant differences (*p* < 0.001) were found for the BDI categories in the MS group compared to the non-MS group (Table 2).

## 4. Discussion

This research studied depression in 58 patients with MS compared to 58 healthy subjects, the first case-control study of this type carried out on the Spanish population. The results obtained showed that many of the people with MS (41.4%) are at risk of suffering from depression in at least some range of the BDI.

According to a recent study, treating depression in MS patients significantly improves fatigue [33]. Therefore, it is important for these patients to be evaluated by a multidisciplinary team in order to improve their quality of life.

Depression tends to be related to other symptoms such as pain and fatigue exacerbated by the psychomotor degeneration of these patients [34]. The prevalence of depression in patients with MS is remarkably high, although it is still not properly diagnosed, leading to cognitive impairment causing the risk of suicide [35]. In the review by Claudio Solaro et al., it is stated that for the correct management of depression in subjects with MS, it is necessary to understand the damage in the central nervous system using MRI and relate fatigue and pain with depression [16].

In agreement with our study, patients with MS (51%) present clinically significant depressive symptoms [36]. Thus, we compared the control group with the case group, taking all the patients without considering depression as an inclusion criterion, and when reviewing the data obtained, we found that many people with MS (19%) present a mild level of depression.

For our study, the range of depression was obtained with the Beck Depression Inventory (BDI) questionnaire. This document was validated by other authors as having optimal characteristics to establish depression in subjects with MS [27], and furthermore, this questionnaire correlates with other types of questionnaires used to study depression, fatigue, and affect (Hamilton Rating Scale, Yale Single Questionnaire and PDQ) in patients with MS [37,38,39]. For this reason, studies conducted on depression in MS tend to have different results due to the use of other types of questionnaires and different types of samples.

Other studies carried out on depression in chronic pathologies also recommend the BDI as a good self-assessment tool for screening and evaluation in clinical practice of depression, its intensity, and its evolution in patients with chronic kidney disease [40]. As depression not only affects patients with MS or other chronic diseases but also their family environment, the BDI proved to be a valid and reliable instrument to measure depression in the family caregivers of children with chronic diseases [41]. Shelby et al., in their study on Parkinson’s disease, showed that the BDI in clinical practice is suitable for additional psychiatric evaluation and for adopting different therapeutic interventions [42]. This agrees with the study by Ana María Jiménez-Cebrián et al. where, for the first time, it was shown in a sample of subjects with Parkinson’s compared to healthy subjects that depression represents a significant potential risk for the increase in symptoms and has a negative impact on these patients compared to healthy subjects [43]. We can, thus, based on previous studies and our results, consider that depression and chronic pathologies have an unwanted effect on the quality of life of people with MS, especially in somatic-vegetative aspects such as fewer hours of sleep, loss of energy, greater tiredness, and loss of appetite [6,24,44].

Taking into account our findings and the bibliographic review, it is necessary for patients with MS to be aware of the importance of depression on their quality of life and to offer them a multidisciplinary team to establish good management of this pathology. In addition, biomarkers have been studied in some research with MS subjects, but all of them refer to the need for a larger sample for better consistency to thus complete the aspects of depressive studies and their possible causes in MS [11,40].

To correctly manage MS and depression, it would be important to use other biomarkers tools (blood or salivary) together with the BDI to provide more consistency and more treatment possibilities for this group of patients, determine the triggers involved, and improve their quality of life.

## 5. Conclusions

The results obtained show us that people with MS have higher scores on all levels of depression compared to healthy subjects, with greater differences at the mild and moderate levels.

## Figures and Tables

**Table 1:** Descriptive and socio-demographic data of the sample.

Demographic and Descriptive Data		Total Group *n*= 116 Mean ± SD (IC95%)	MS *n* = 58 Mean ± SD (IC95%)	Healthy *n*= 58 Mean ± SD (IC95%)	*p* Value *
Age (Years)		47.38 ± 10.62 (24–66)	47.38 ± 10.68 (24–66)	47.38 ± 10.65 (24–66)	1.000 †
Weight (kg)		71.36 ± 12.75 (46–105)	70.19 ± 13.31 (46–105)	72.53 ± 12.16 (47–100)	0.324 †
Height (cm)		167.68 ± 8.43 (150–188)	167.24 ± 8.49 (150–183)	168.12 ± 8.43 (154–188)	0.577 †
BMI (Kg/m^2^)		25.32 ± 4.14 (18.0–37.5)	24.98 ± 3.94 (18.0–37.5)	25.65 ± 4.34 (18.4–37.2)	0.391 †
Time since MS diagnosis (years)		N/A	12.55 ± 8.53 (1–33)	N/A	<0.001 †
Sex (%)	Male Female	34 (29.3 %) 82 (70.7 %)	17 (29.3 %) 41 (70.7 %)	17 (29.3 %) 41 (70.7 %)	1.000 ‡

Comparison of the demographic characteristics of the total sample, MS with foot pain and healthy matched MS with normalized reference values BMI: Body mass index; * Mean ± standard deviation, range (min-max), and Student’s *t* test were applied for independent samples. In all analyses, *p* < 0.05 (with a 95% confidence interval) was considered statistically significant and † Student’ *t*-test was applied for independent samples. ‡ The Chi-square test was used.

**Table 2:** Relationship of scores and categories of the BDI between patients with MS and healthy controls.

Outcome Measurements		Total Group Mean ± SD (*n* = 116)	Cases Mean ± SD (*n* = 58)	Controls Mean ± SD (*n* = 58)	*p*-Value (Cases vs. Controls)
BDI Category *	No	82 (70.7%)	34 (58.6%)	48 (82.8%)	0.022 *
	Mild	18 (15.5%)	11 (19%)	7 (12.1%)	
	Moderate	12 (10.3%)	10 (17.2%)	2 (3.4%)	
	Severe	4 (3.4%)	3 (5.2%)	1 (1.7%)	
BDI scores		7.28 ± 6.90 (0–28)	9.52 ± 7.70 (0–28)	5.03 ± 5.14 (0–24)	0.001 †

* BDI, Beck depression inventory. Frequency, percentage (%), and Chi-square test (x^2^) were utilized. BDI domains were divided as follows: (1) 0 to 9 points: Without depression, (2) 10 to 15 points: Mild depression, (3) 16 to 23 points: Moderate depression, and (4) 24 to 57 points: Severe depression. † BDI scores, Median ± interquartile range, range (min–max), and Mann–Whitney U test were used. In all the analyses, *p* < 0.05 (with a 95% confidence interval) was considered statistically significant (bold).

## Data Availability

The dataset supporting the conclusions of this article is available upon request to f.ruiz@udc.es in the Research, Health and Podiatry Group, Department of Health Sciences, Faculty of Nursing and Podiatry. Industrial Campus of Ferrol, Universidade da Coruña, 15403 Ferrol, Spain.
